# TEMPO-Functionalized Nanoporous Au Nanocomposite for the Electrochemical Detection of H_2_O_2_

**DOI:** 10.1155/2018/1710438

**Published:** 2018-06-10

**Authors:** Dongxiao Wen, Qianrui Liu, Ying Cui, Huaixia Yang, Jinming Kong

**Affiliations:** ^1^Pharmacy College, Henan University of Chinese Medicine, Zhengzhou 450008, China; ^2^School of Environmental and Biological Engineering, Nanjing University of Science and Technology, Nanjing 210094, China

## Abstract

A novel nanocomposite of nanoporous gold nanoparticles (np-AuNPs) functionalized with 2,2,6,6-tetramethyl-1-piperidinyloxy radical (TEMPO) was prepared; assembled carboxyl groups on gold nanoporous nanoparticles surface were combined with TEMPO by the “bridge” of carboxylate-zirconium-carboxylate chemistry. SEM images and UV-Vis spectroscopies of np-AuNPs indicated that a safe, sustainable, and simplified one-step dealloying synthesis approach is successful. The TEMPO-np-AuNPs exhibited a good performance for the electrochemical detection of H_2_O_2_ due to its higher number of electrochemical activity sites and surface area of 7.49 m^2^g^−1^ for load bigger amount of TEMPO radicals. The TEMPO-functionalized np-AuNPs have a broad pH range and shorter response time for H_2_O_2_ catalysis verified by the response of amperometric signal under different pH and time interval. A wide linear range with a detection limit of 7.8 × 10^−7^ M and a higher sensitivity of 110.403 *μ*A mM^−1^cm^−2^ were obtained for detecting H_2_O_2_ at optimal conditions.

## 1. Introduction

Hydrogen peroxide (H_2_O_2_) is the smallest and simplest peroxide, which could be generated in many biological processes and cause severe oxidative damage [1–3]. It has been applied in many biosynthetic reactions and also plays an important role in various fields, especially in immune cell activation, vascular remodelling, apoptosis, stomatal closure, root growth, and so on [1, 4]. Because of this, many practical explorations have been done in the detection and monitoring of H_2_O_2_ in pharmaceutical [1–6], biological [5, 6], clinical [1, 3, 7], chemical, textile [1, 2, 8–10], and food industries [1–6, 10]. The dynamic equilibrium of the production and consumption of H_2_O_2_ is closely interlinked with the quality of our life; therefore, the development of new materials and techniques for quantitative detection of H_2_O_2_ at a trace level has presented a significant role in the fundamental studies of diagnostic and monitoring applications. However, most existing techniques suffer from many drawbacks, such as inherent instability, time consuming, poor selectivity, low activity, complicated, and costly immobilization procedures [1–3, 9–11]. Thus, it is necessary to develop new electrochemical sensors overcoming those drawbacks for accurate and sensitive detection of H_2_O_2_.

A number of new materials such as metals nanomaterials, carbon nanotubes, quantum dots, nanocomposites, and redox substances have been employed for the detection of H_2_O_2_ [1–9]. In recent studies, nanoscale hollow materials have attracted scientists' attention for their unique porous structural and versatile properties. There have been some reports already about its applications in fuel cell and remarkable catalytic oxidation activities to CO, methanol, hydrazine, and so on [12–15]. Particularly, nanoporous gold nanoparticles (np-AuNPs), one of the most important nanoscale hollow materials, combining their high surface areas with tunable surface plasmon resonance (SPR) features, can serve larger immobilized surface and excellent catalytic sites in chemical reactions and also provide a substrate material to manufacture functionalized nanocomposite [12, 15]. Meanwhile, TEMPO plays a key role in chemistry and biology as an organic redox catalyst for alcohol, aldehydes, and ketones [16–21] and has shown its catalytic oxidation potential to H_2_O_2_ due to the electrochemical oxidation of stable nitroxyl radical [22, 23]. Recently, the nitroxyl radical of TEMPO has been reported to attach to solid surfaces by chemical routes to form supramolecular assemblies with high coverage of catalytic radicals [24, 25]. Based on considerations above, in order to take full advantage of their inherent catalytic oxidation activity of the np-AuNPs and the TEMPO, we explore the synergistic effect of TEMPO-functionalized np-AuNPs for the peroxidase-like activity response and use this feature for H_2_O_2_ detection.

Most nanoporous materials are prepared by using traditional dealloying, template, electrochemical methods, and directed self-assembly [9, 10, 26], and the dealloying approach is mostly adopted to synthesize various nanoporous hollow structures [12–15], using AgCl templates to get the nanoporous gold [12, 26, 27]. However, this traditional dealloying approach is limited by its harsh cumbersome and high-heat conditions [12, 28]. In this work, we prepared the zero-dimensional hollow np-AuNPs via an improved one-step dealloying synthesis in moderate conditions instead of traditional two-step dealloying methods. To improve the peroxidase-like activity of the np-AuNPs and TEMPO, we assembled mercaptoacetic acid (MA) on the surfaces of np-AuNPs/GCE, and then the 4-carboxy-TEMPO was connected to the mercaptoacetic acid through Zr^4+^ as the bridge bond. The np-AuNPs were characterized by scanning electron microscopy (SEM) and UV-Vis spectroscopy, and the TEMPO-contained nanocomposites characterized its electrochemical active area by cyclic voltammetry in different electrolytic buffer. Finally, its amperometric response of TEMPO-np- AuNPs/ GCE to detect H_2_O_2_ was exploited.

## 2. Experimental

### 2.1. Chemical Reagents

Silver nitrate (99%), HAuCl_4_·3H_2_O (>99.9%), hydroquinone (>99%), H_2_O_2_ (30wt% aqueous), and mercaptoacetic acid (MA) were obtained by Sinopharm Chemical Reagent Co., Ltd. (Beijing). The polyvinylpyrrolidone (PVP, MW 1,300,000) was purchased from J&K Scientific Ltd. Zirconium dichloride oxide octahydrate (ZrOCl_2_·8H_2_O) and 4-carboxy-2,2,6,6-tetramethylpiperidine-1-oxyl free radical (4-carboxy-TEMPO) came from Sigma-Aldrich (St. Louis, MO) and TCI (Shanghai) Development Co., Ltd., respectively. 0.1 M PBS supporting electrolyte was prepared by orthophosphoric acid and its salts (0.1 M Na_2_HPO_4_, 0.1 M NaH_2_PO_4_), pH=4.0-9.0. All reagents were of analytical grade.

### 2.2. Apparatus

A scanning electron microscopy (SEM, Hitachi S-4800, Japan) was employed to study the morphology of nanoporous gold nanoparticles and TEMPO-MA-np-AuNPs on the electrode. The UV-Vis absorption peak was carried on a UV-3600 spectrophotometer (SHIMADZU). All data of electrochemical studies were obtained with an electrochemical workstation (CHI 760D, Chenhua, Shanghai) at the room temperature. A platinum wire auxiliary electrode, a saturated calomel reference electrode, and a modified glassy carbon electrode (GCE, Φ=3 mm, as the working electrode) are included in a standard three-electrode cell. All ultrapure water (≥18.25 MΩ) for experiments was obtained from a Millipore Milli-Q water purification system.

### 2.3. Preparation of Nanoporous Gold Nanoparticles

The nanoporous gold nanoparticles (np-AuNPs) were prepared according to the literature with some modification [12]. Briefly, 160 *μ*L solution of hydroquinone (28 mM) and 10 mM AgNO_3_ aqueous solution (60 *μ*L) were mixed firstly into 4.5 mL PVP solution (90 mM) in sequence. Then the mixed system came to an equilibrium stirring for 5s, 650 r.m.p. Next, 40 mM HAuCl_4_ (100 *μ*L) was added dropwise into the system at room temperature under gentle stirring. After 3 min standing, the color of the reaction liquid turned to stable (reddish brown), and then the residual AgCl, which is formed during the reaction, was removed by the additional concentrated NH_4_OH (1 mL). Finally, the resulting solution was centrifuged and washed repeatedly with ultrapure water, 7,000 r.p.m. 5 min, to collect the np-AuNPs. Finally, 1.0 mg of np-AuNPs sample was fully suspended in a mixed solution (1.0 mL ethanol, 1.0 mL of 90 mM PVP) by ultrasonication.

### 2.4. Electrode Fabrication of Tempo-Np-Au NPs/GCE

The bare glassy carbon electrode (GCE) was polished with 0.30*μ*M Al_2_O_3_ slurries on the chamois leather until a mirror-like surface is obtained. Next, it was ultrasonically cleaned with absolute ethanol and ultrapure water, dried with N_2_. Then, the electrode was subjected to cyclic voltammetry (CV) in 0.1 M KCl with the potential of -0.4V and 1.6 V, at a scan rate of 100 mVs^−1^, until the reproducible cyclic voltammograms were obtained.

**Figure 1:**
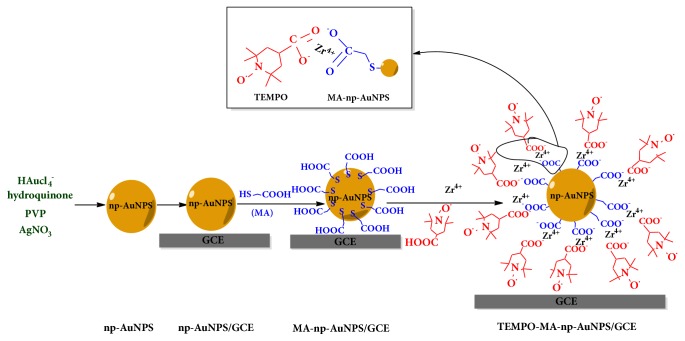
Preparation of TEMPO-MA-np-AuNPs/GCE for the electrochemical determination of H_2_O_2_.

A quantity of 1*μ*L of the prepared np-AuNPs suspension was dropped on the surface of freshly pretreated bare GCE above. After the GCE was air dried at room temperature, 20 *μ*L of 0.2 mM MA solution was added onto the np-AuNPs/GCE surface and incubated for 1.0 h at room temperature; by so doing, a MA self-assembled monolayer formed on the surface of np-AuNPs (MA-np-AuNPs/GCE) via chemisorption and the chemistry of formation of MA-SAM on the np-AuNPs surface; a procedure probably involves the oxidative addition to form the S-Au bond by losing the hydrogen as H_2_ or H_2_O [29, 30]. Subsequently, the TEMPO-np-AuNPs/GCE was prepared by carboxylate- zirconium-carboxylate chemistry [31–33]. After washing the MA-np-AuNPs/GCE with ultrapure water to remove the remaining MA, the electrode was immersed in ZrOCl_2_·8H_2_O 60% ethanol solution (5.0 mM) for 30 min. The modified electrode was taken out and then washed with absolute ethanol, dried with N_2_. Next, the electrode was incubated in 20 *μ*L of 0.2 mM 4-carboxy-TEMPO solution for 30 min, followed by rinsing with ultrapure water to remove remaining reactants. TEMPO-functionalized nanoporous Au nanocomposite electrode was prepared. The establishment of this sensor for H_2_O_2_ detection is depicted in Figure 1.

## 3. Results and Discussion

### 3.1. Characterization of Modified Electrode by SEM and UV-Vis

**Figure 2:**
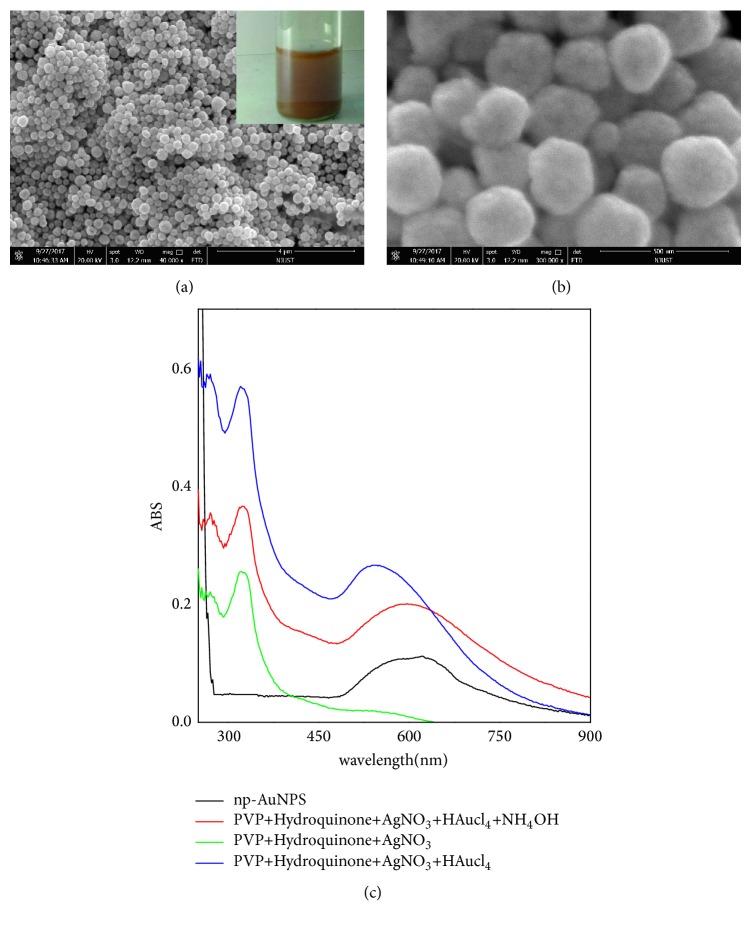
SEM images (a and b) of nanoporous gold nanoparticles (np-AuNPs) and typical UV-Vis absorption spectra (c) of the reactions during the prepared process of np-AuNPs.

By following procedures above, we successfully synthesized the free-standing np-AuNPs under moderate conditions by using a one-step aqueous solution-based approach, which circumvents the limits of stringent and harsh multistep protocols of traditional dealloying approaches. As shown in Figures 2(a) and 2(b), SEM images indicate that the as-synthesized np-AuNPs have a shape of spherical and exhibit an extremely roughened surface, which is consistent with the result of Srikanth's report [12].

As it has been known to all, AgNPs and AuNPs including np-AuNPs are attractive due to their surface plasmon resonance (SPR) properties. Ag^+^ AuNPs and np-AuNPs often exhibit different spectral absorptions in the UV-Vis wavelength region [26, 34]. Therefore, we can use absorption spectroscopy to monitor the process of reaction; the UV-Vis spectral absorptions of the as-prepared np-AuNPs are shown In Figure 2(c). A peak appeared at 324 nm, which is the typical UV-Vis absorbance peak of Ag^+^ [34]. After 100*μ*L HAuCl_4_ solution was added in the system, a significant increase of signal appeared at 530 nm, which is caused by the grown of AuNPs on the surface of AgCl templates in the kinetic-controlled process [35]. Subsequently, with NH_4_OH added to stop the reaction, the surface plasmon resonance (SPR) peak of 530 nm underwent a red shift to 596 nm-700 nm with longer and broader profile, which belongs to the characteristic peak of porous gold nanoparticles. The typical SPR peak of AuNPs always exhibited around 530 nm; the Ag° broad peak maximum usually occurs at 410 nm, in solution [34]. It was found that, after the centrifuge step to remove residual AgCl, the absorbance peak at 324 nm disappeared, indicating the formation of the np-AuNPs.

### 3.2. Characterization of Modified Electrode by Cyclic Voltammetry

**Figure 3:**
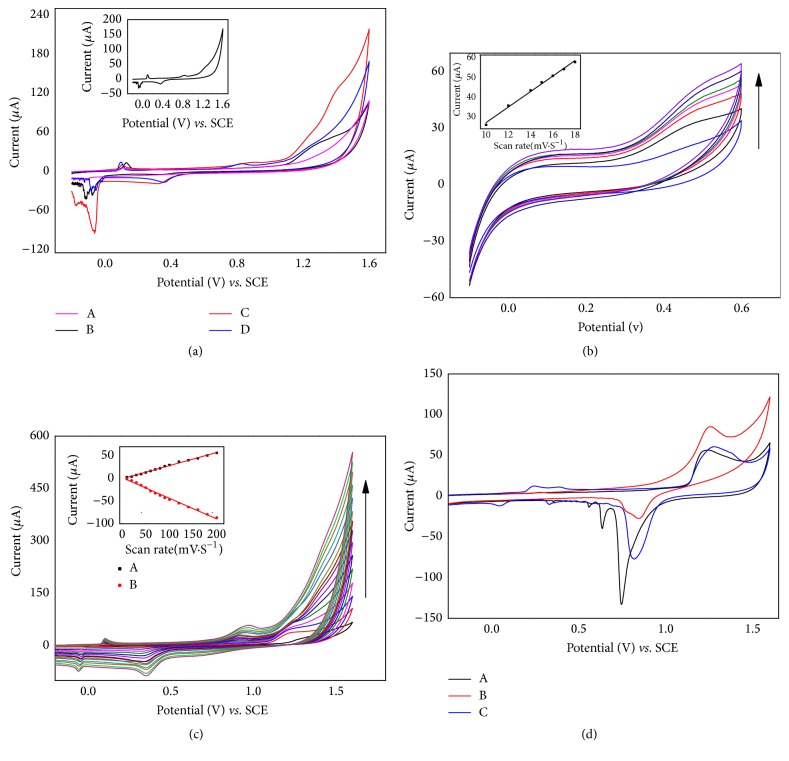
** (a)** Different modified GCE in 0.1 M PBS (pH = 7.0) under N_2_. Scan rate: 50mVs^−1^. (A) Bare GCE, (B) np-AuNPs/GCE, (C) MA-np-AuNPs/GCE, and (D and inset) TEMPO-np-AuNPs/GCE. CVs of np-AuNPs** (b)** and TEMPO-np-AuNPs (**c)** on the GC electrode surface under N_2_, 0.1 M PBS (pH = 7.0) at different scan rates from 10mVs^−1^ to 200mVs^−1^. Inset: (**b**) the linear relationship between the scan rate and the currents at a potential of 0.55V. (**c**) The linear relationship between anodic (black spots, at 0.95 V) and cathodic (red spots, at 0.38 V) peak currents and scan rate.** (d) **CVs of different electrodes in 0.5 M H_2_SO_4_ under N_2_, 0.1 M PBS (pH = 7.0) at a scan rate of 100 mVs^−1^.(A) np-AuNPs, (B) MA-np-AuNPs, and (C) TEMPO-np-AuNPs.

To characterize the modified electrode TEMPO-np-AuNPs, the typical cyclic voltammetry was performed. The cyclic voltammogram (CV) curves of different modified electrodes in the absence of oxygen in 0.1 M PBS supporting electrolyte (pH = 7.0), at 50 mVs^−1^, are shown in Figure 3(a). For the bare GCE, there are no redox peaks (A). With the np-AuNPs added on the GCE surface, a broad peak arose at 1.2-1.5V (B) due to the increased effective electroactive area of np-AuNPs. What is more, when MA self-assembled on the np-AuNPs, an obvious peak increase was observed due to the S-Au bond formation (C). By comparing the CVs of C and D, a new pair of well-behaved redox peaks of 0.38 V (cathodic peak) and 0.82 V (anodic oxidation peak) was obtained after 4-carboxy-TEMPO was finally attached to the former-electrode surface. In addition, the decreases of a pair of peaks at -0.1 V/1.2-1.5 V due to the steric hindrance also indicted that 4-carboxy-TEMPO was successfully connected with MA-np-AuNPs via carboxylate-zirconium-carboxylate chemistry.

Further, we characterized the formed np-AuNPs with electroactive activity through CV at different scan rates from 10 mVs^−1^ to 200 mVs^−1^. As shown in Figure 3(b), the intensities of the electric current increased linearly along with the increase of scan rates (*k*_1_ = 3.934 (± 0.122)), which confirmed that the electroactive np-AuNPs attached on the GCE electrode surface by adsorption according to the theory of homogeneous redox catalysis [22], rather than other interactions. As mentioned above, the CV of TEMPO-np-AuNPs has a new pair of redox peak (0.38 V/0.82 V), while the CV of np-AuNPs did not. As displayed in Figure 2(c), with the scan rates increasing, the current intensity of this redox couple enhanced with a slight redshift; both the I_pa_ (black spots) and I_pc_ (red spots) peak currents were linearly proportional to the scan rate. Their slopes, respectively, were *k*_pa_ = 0.290 (± 0.005) and *k*_pc_ = -0.456 (± 0.011). Those behaviors illustrate that the modified np-AuNPs (TEMPO-np-AuNPs) had the adsorption with the GCE electrode surface, which are in accordance with the np-AuNPs and literatures [42, 43].

### 3.3. Electrochemical Measurement of Np-AuNPs Active Surface Areas

The effective areas of different surface modification were estimated by CV method. As shown in Figure 3(d), the anodic oxidation current of the three curves all rose at about 1.2 V and had a typical reduction peak around 0.75 V, which is caused by the reversible redox reaction in 0.5 M H_2_SO_4_. However, the MA-np-AuNPs (B) exhibit a higher peak at 0.75 V and sustain a large redshift as compared with np-AuNPs (A), indicating that it is hard to be oxidized with H_2_SO_4_ due to the increased impedance of charge transfer after MA is immobilized on the surface of np-AuNPs. When the TEMPO is chemically modified on MA-np-AuNPs (C), the cathodic peak (around 0.75 V) had an obvious enhancement, which may be caused by the diffusion layer of TEMPO· and is a three-dimensional steady-state. Besides, the rough porous surface of np-AuNPs contributed to generate the multimodal of the curve (e.g., 0.20 V-0.45 V of curve C).

For each experiment, the amount of np-AuNPs used was the same; CV of np-AuNPs in 0.5 M H_2_SO_4_ (A) were measured to calculate their electroactive surface areas via integrating the area of the gold oxide reduction curve. An electroactive surface area of 7.49 m^2^g^−1^ for np-AuNPs is obtained by Randles-Sevcik equation and assuming a specific charge of 450 *μ*C cm^−2^ for the gold oxide reduction [12, 44]. Notably, the active surface areas of np-AuNPs are higher than that of the commercial Au electrodes, Au nanoparticle, Au nanocoral, and other kinds of Au electrodes [45], near 49 times high according to report. The large surface-to-volume ratio of metal nanoparticles is closely related to its high electrical conductivity, catalytic ability, and surface reaction activity such as for the detection of H_2_O_2_ [2, 45].

### 3.4. Electrochemical Detection of H_2_O_2_

**Figure 4:**
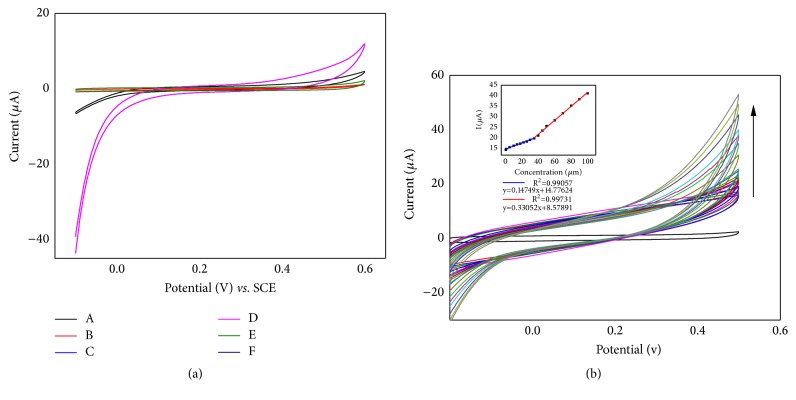
**(a)** CVs of electrodes in the absence (A-C) and presence (D-F) of H_2_O_2_ (3mM) in N_2_-saturated 0.1 M PBS (pH = 7.0). Scan rate: 20mVs^−1^. (A and E) Bare GCE (curves B and F) AuNPs/GCE (curves C and D) np-AuNPs/GCE.** (b)** CVs of np-AuNPs/GCE in 0.1 M PBS (pH = 7.0) with N_2_ toward different concentrations of H_2_O_2_ over the range of 0.5 *μ*M to 100 *μ*M. Applied potential: 0.55 V. Inset: plot of electrocatalytic current of H_2_O_2_ versus its concentrations.

As we have known already, the electrochemical behavior of np-AuNPs modified GCE electrode for H_2_O_2_ was studied by CV. As shown in Figure 4(a), when 3 mM H_2_O_2_ was added to 0.1 M phosphate buffer saline (pH=7.0) under N_2_, compared to the bare GCE, distinct increases of the AuNPs/GCE and np-AuNPs/GCE response currents were observed. Interestingly, the np-AuNPs/GCE had a significant advantage in the difference of the current intensity (ΔI) under the same condition in the presence of H_2_O_2_. As one of the most famous catalytic materials, Au nanomaterials with different shapes and structures have been suggested good responses for electrocatalytic H_2_O_2_ [1–3].The current responses toward H_2_O_2_ concentration over the range of 0.5 *μ*M-100 *μ*M on the np-AuNPs/GCE (Figure 4(b)) were studied. The current intensity had a steady rise with the increasing of H_2_O_2_ concentration. The HO· radical resulted from H_2_O_2_ would be stabilized by the np-AuNPs [3, 46]. The surface property of np-AuNPs may influence the catalytic ability of H_2_O_2_ and the charge-transfer processes. When the H_2_O_2_ concentration is below 26 *μ*M, it exhibited a linear correlation of I (*μ*A) = 0.14749 C (*μ*M) +14.77624 (R^2^ = 0.99057, RSD = 4.2%) between 0.5 *μ*M and 26 *μ*M. With the increase of stable surface charge transfer for the continuous regeneration of charge-transfer complex [47], it may fill the concave surface of np-AuNPs, and the slope (*K*_b_) of the oxidation catalytic linear response grew to 0.3305 (R^2^ = 0.9973) in the range of 26 *μ*M-100 *μ*M. Results of np-AuNPs /GCE show a good catalytic detection activity to H_2_O_2_.

**Scheme 1:**
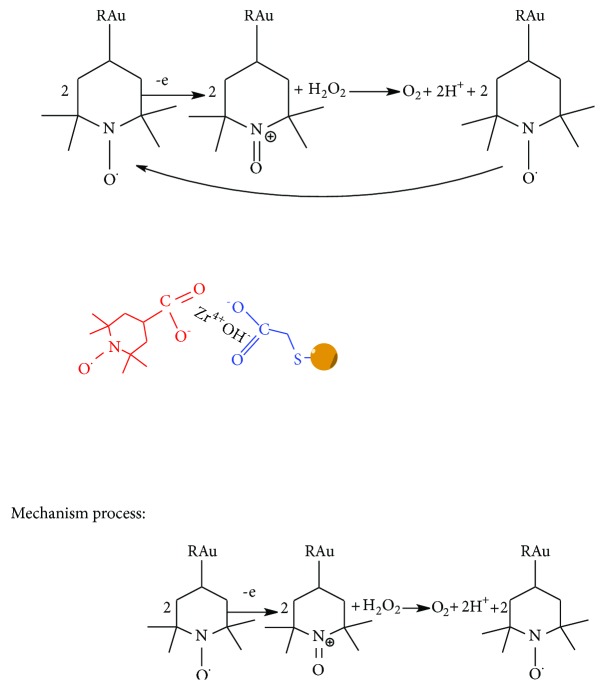
Equilibrium of single electron between TEMPO-np-AuNPs and H_2_O_2_.

**Figure 5:**
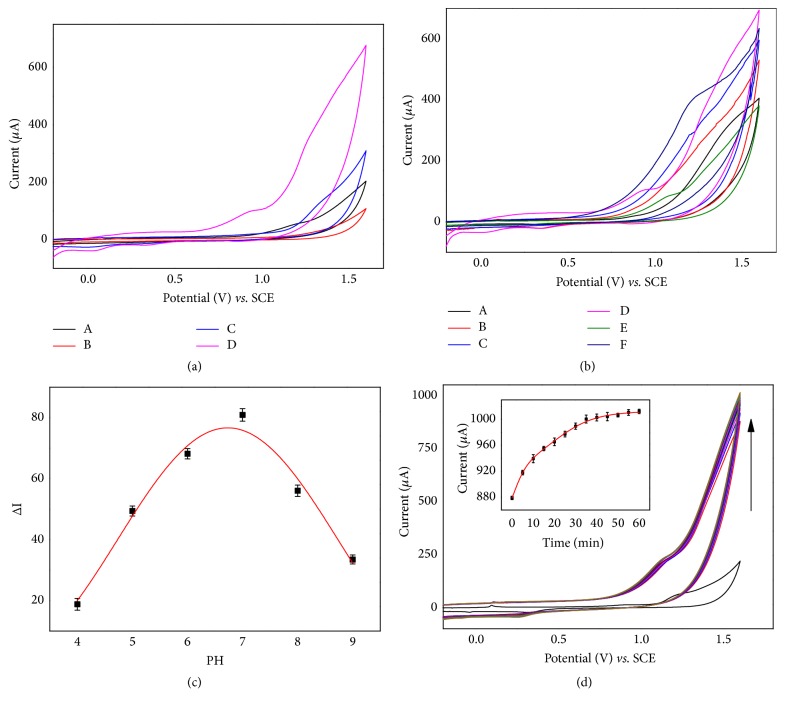
**(a)** CVs of different GCE under the absence (A and B) and presence (C and D) of 5mM H_2_O_2_ in N_2_-saturated 0.1 M PBS (pH = 7.0) at a scan rate of 50mVs^−1^. (A) Bare GCE (B and C) np-AuNPs/GCE, and (D) 4-carboxy-TEMPO-np-AuNPs/GCE. Optimization of experimental conditions:** (b)** CVs of the TEMPO -np-AuNPs/GCE in different pH values at the range of 2.0~9.0 and** (c)** the relationship between the net current at 0.95V with different pH.** (d)** CVs of TEMPO-np-AuNPs/GCE in 0.1 M PBS (pH = 7.0), in the presence 5 mM H_2_O_2_, in different times from 0 min to 60 min, N_2_-saturated, 50mVs^−1^. Inset: the changes of the electric current (1.6 V) with the time increasing.

CV voltammograms of different product on GC electrode in the absence and presence of hydrogen peroxide were shown in Figure 5(a). When H_2_O_2_ was put into 0.1 M PBS electrolyte buffer, an obvious increase of the peak current at 0.95 V in the CV of TEMPO-np-AuNPs is observed unlike that of smooth curves in other CVs. To ascertain the synergistic peroxidase-like activity between the TEMPO and np-AuNPs, we compared the net current strengths (ΔI) of TEMPO-np-AuNPs and np-AuNPs, where the ΔI refers to the difference strength value within H_2_O_2_ in and without it. The nitroxide mediator can improve the free diffuse state, which is adjacent to the electrode surface [22, 23]. The ΔI of np-AuNPs sharply rose to 403 *μ*A after it was modified with the TEMPO. All of these manifest that TEMPO-np-AuNPs have a higher potential peroxidase-like activity to H_2_O_2_. Usually, the H_2_O_2_ biosensor is constructed via the transfers of the two consecutive single electron transfers (Scheme 1) [22]. First of all, the TEMPO-np-AuNPs undergo a stable reversible one-electron oxidation to produce the intermediate at the TEMPO-functionalized electrode. Finally, the intermediate provides an electrocatalytic electron transfer way to detect H_2_O_2_.

### 3.5. Factors Influencing Detection

We all know that time and pH can directly affect the stability of the reaction proceeding and catalytic of enzymes. The analytical performance of the sensor is usually closely linked with the stability of the materials on electrode, which was partially influenced by the time and pH of electrolyte solution. So the pH and time were tested for their electrical catalytic activity to an optimal condition in this work. Firstly, the effect of pH on the potential of electrocatalytic activity on the TEMPO-np-AuNPs was tested at the range of 2.0 to 9.0 under the presence of 5 mM H_2_O_2_.

As can be seen in Figures 5(b) and 5(c), there is a slight negative shift in the potential catalytic site due to the reversible anodic oxidation of nitroxide derivatives and the inherent peroxidase-like activity response to H_2_O_2_ of np-AuNPs. Besides, the current intensity at 0.95V has an obvious enhancement under acidic environment and a distinct reduction followed when it was substituted with alkaline buffer (pH > 7.0). Briefly, TEMPO-np-AuNPs can reach the highest catalytic activity to H_2_O_2,_ which is consistent with the result of the one-electron behavior of TEMPO/TEMPO^+^ [22], under similar physiological conditions (0.1 M PBS, pH 7.0). Results show that pH can inappreciably influence the catalytic activity of TEMPO-np-AuNPs at the range of 5.0 to 8.5. Compared with the pH decided enzyme-modified electrode detection methods, this sensor can advance the application of TEMPO-np-AuNPs to detect H_2_O_2_* in vivo* measurements [1].

Further, we investigated the time factor in the presence of 5 mM H_2_O_2_.  Figure 5(d) shows TEMPO-np-AuNPs can catalyze H_2_O_2_ to produce O_2_, immediately, which then comes to an equilibrium state with a maximum current strength in 40 min. By comparing the current strength at 1.6 V, we found that the floating electric potential of 40 min only takes 13.64% in the whole catalytic oxidation process. It is possible that this remaining increase is closely related to the irregular gaps and surface of the np-AuNPs, which may lead to the time retardation to the reversible one-electron behavior of the redox couple TEMPO/TEMPO^+^ [46]. So we selected 40 min stirring constantly after H_2_O_2_ was added to the 0.1 M PBS with the buffer system of pH=7.0 to ensure the sufficient current response in the following measurements.

### 3.6. Steady-State Amperometric Response of H_2_O_2_

**Figure 6:**
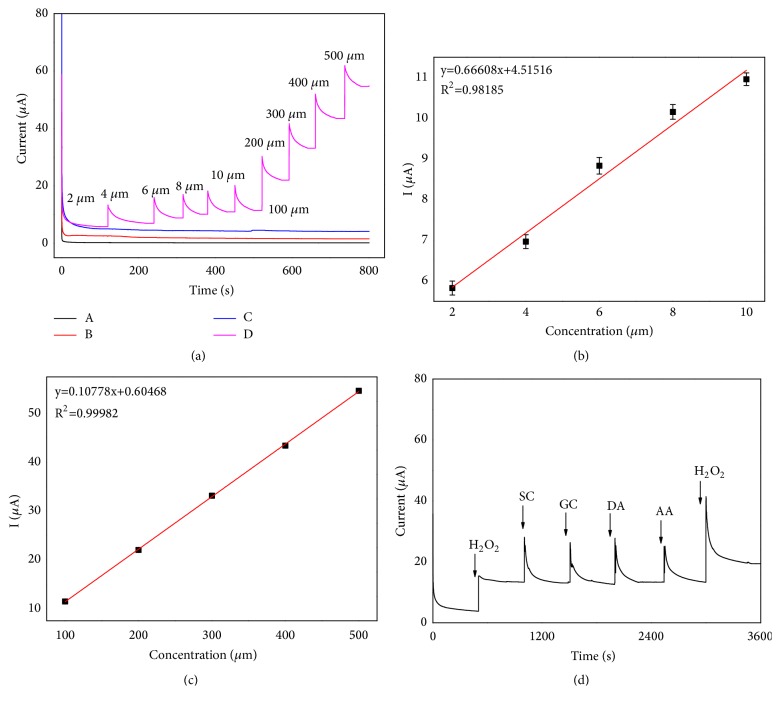
(**a)** Chronoamperometric responses observed at (A) bare GCE, (B) np-AuNPs/GCE, (C) TEMPO-np-AuNPs/GCE, and (D) TEMPO-np-AuNPs/GCE after successively injecting H_2_O_2_, in 0.1 M PBS (pH=7.0). Applied potential: 1.10 V and the calibration plot between the oxidation current and the H_2_O_2_ concentration** (b and c). **(**d)** Amperometric response of H_2_O_2_ and interferants at TEMPO-np-AuNPs/GCE at 1.10V in PBS (0.1 M, pH = 7.0). Injection sequence: 0.1mM H_2_O_2_, 100 mM SC, 100 mM GC, 100 mM DA, 100 mM AA, and 0.2 mM H_2_O_2_.

To evaluate the applied potential of TEMPO-MA-np-AuNPs/GCE as peroxidase-like. We investigated the response of the amperometric signal under the optimal condition with the 0.95 V as the applied potential (*E*_app_). As shown in Figure 6, the TEMPO-np-AuNPs/GCE not only can achieve a quick steady-state current within 10 s, but also has a stronger response (C) compared to the np-AuNPs/GCE (B).

Moreover, with the addition of same amount H_2_O_2_ in every interval of 100 s, the TEMPO- np-AuNPs nanocomposite on the GCE exhibited a good linear chronoamperometric response to H_2_O_2_ from 2.0 *μ*M to 500 *μ*M, which is shown in Figure 6. As what we have seen, when the concentration of H_2_O_2_ is below 10.0 *μ*M, the slope of the linear (*K*_*b*_= 0.66608,* R*^2^=0.98185) is lower than that in a higher concentration range. In other words, the sensitivity of electrocatalytic oxidation activity to H_2_O_2_ is improved with the concentration of H_2_O_2_ increased in the solution and there are no substrate inhibition effects occurring at high concentration of H_2_O_2_. With successive addition of H_2_O_2_ (n = 5) as shown in Figures 6(b) and 6(c), we can obtain two linear regression equations: (1) below 10.0 *μ*M, y = 0.66608x+4.51516 (*R*^2^ = 0.98185); (2) 10.0 *μ*M to 500 *μ*M, y = 0.1078x+0.6047(*R*^2^ = 0.9998). Error bars represent the standard deviations of five independent measurements. The repeatability of the system is assured by a relative standard deviation (RSD) of 2.8%. We use conventional three times the standard deviation of [LOD = 3(RSD/slope)] to estimate the limit of detection (LOD) of H_2_O_2_, which is 0.78 *μ*M in our work and lower than some Au nanomaterials and nitroxide derivatives for H_2_O_2_ detection as listed in Table 1. The results of the stability measurements indicated that np-AuNPs still keep its stable original roughened surface and porous structure after 30 days of storage at 4°C. Moreover, compared with previous results, the TEMPO-np-AuNPs/GCE can retain 98.6% of its initial current response results in the same measurement conditions. Therefore, the TEMPO-np-AuNPs nanocomposite has a remarkable superiority for the electrochemical detection of H_2_O_2_ over the conventional electrochemical sensing materials and most of the reported Au nanomaterials and nitroxide derivatives probes (Table 1).

### 3.7. Interference Study

Some coexisting potential electroactive species may affect the sensor response, such as sucrose (SC), glucose (GC), dopamine (DA), and ascorbic acid (AA) [3, 48]. Good selectivity is crucial to ensure and facilitate the accurate assessment for biosensor in a particular application. For better detection* in vivo*, the interference study of TEMPO-np-AuNPs/GCE for the electrochemical detection of H_2_O_2_ was carried out to evaluate its practical feasibility. Results were shown in Figure 6(d); a discernible slight fluctuation is hard to see after the abundant successive addition of each interfering species (SC/GC/DA/AA), while no obvious interference signal was observed. Notably, the TEMPO-np-AuNPs/GCE had an 18*μ*A response (*E*_app_ = + 0.95 V) as soon as another 0.1mM H_2_O_2_ was injected into the complex system of interference. The initial small responses caused by SC, GC, DA, and AA belong to normal current fluctuations, which is a deductible interference compared with that caused by H_2_O_2_. TEMPO-np-AuNPs/GCE exhibited an acceptable selectivity towards the practical* in vivo* electrochemical detection of H_2_O_2_.

## 4. Conclusions

In this work, we demonstrated a strategy for producing large specific surface area nanoporous gold nanoparticles and manufactured the TEMPO-functionalized np-AuNPs nanocomposite; the electrooxidation to H_2_O_2_ with a high density of radicals on the TEMPO-np-AuNPs surface was also investigated. It is worth noting that this new nonenzymatic H_2_O_2_ probe is prepared under a gentle, secure, low-cost, and simple procedure. When the np-AuNPs are combined with 4-carboxy-TEMPO, the advantages of their unique properties for the electrochemical detection of H_2_O_2_ come to a double effective enhancement. Compared the peroxidase-like activity based on the direct electron transfer of the TEMPO-np-AuNPs/GCE to the electrochemical hydrogen peroxide biosensors of TEMPO-based ligand, we obtained a wide linear range for H_2_O_2_ detection. The enzyme-like activity with low detection limit, high sensitivity, low-cost, anti-interference, good reproducibility, and stability of this nanocomposite with TEMPO-based ligand make a contribution to improve the detection current signal of H_2_O_2_. Furthermore, this TEMPO-functionalized np-AuNPs nanocomposite study plays a significant role in facilitating the research of biosensors, gold nanomedicine, catalysis, or cancer therapy.

## Figures and Tables

**Table 1:** Comparison of recent Au nanomaterials and nitroxide derivatives for H_2_O_2_ detection.

Electrode design	L. R. (M)	D.L. (*μ*M)	Stability	Reference
HRP/Cys/AuNP/ITO	8.0×10^−6^~3.0×10^−3^	2.00	83% (12 weeks)	[6]
HRP/CaCO_3_-AuNPs/ATP/Au	5.0×10^−7^~5.2×10^−3^	0.10	96.4% (30 days)	[36]
HRP-nano-Au	1.2×10^−5^~1.1×10^−3^	6.10	75% (5 weeks)	[37]
HRP/AuNPs/poly(St-co-AA)	8.0×10^−6^~7.0×10^−3^	4.00	97.8 % (60 days)	[38]
Au/CeO_2_ nanocomposit*e*	0~3.0×10^−4^	5.00	-* *-* *-	[9]
AuNPs-N-GQDs/GC	2.5×10^−5^~1.3×10^−2^	0.12	89%, 3 weeks	[10]
Poly(BCB)/Au-NPs/GCE	6.0×10^−5^~1.0×10^−2^	0.23	95 %(2 week)	[39]
GC/MTMOS-Au^73^Ag^27^	1.0×10^−5^~7.0×10^−5^	1.00	-* *-* *-	[40]
Hb/Au nanoflowers/CNTs/GCE	1.0×10^−6^~6.0×10^−4^	7.30	-* *-* *-	[41]
TEMPO/GCE	1.0×10^−7^~1.0×10^−8^	0.05	100%,3 months	[22]
ChOx/TEMPO/GCE	2×10^−5^~ 2.5 ×10^−3^	20.0	-* *-* *-	[23]
TEMPO-MA-np-AuNPs/GCE	2.5×10^−6^~5.0×10^−4^	0.78	98.6%(30 days)	**This work**

## Data Availability

All the data used to support the findings of this study are included within the article or are available from the corresponding author upon request. These data are available for unrestricted use, distribution, and reproduction in any medium, provided the original work is properly cited.
